# The Role of Negative Affect in Emotional Processing of Food-Related Images in Eating Disorders and Obesity

**DOI:** 10.3389/fpsyg.2021.723732

**Published:** 2021-08-23

**Authors:** Irene Sierra, Cristina Senín-Calderón, María Roncero, Conxa Perpiñá

**Affiliations:** ^1^Departamento de Personalidad, Evaluación y Tratamientos Psicológicos, Universitat de València, Valencia, Spain; ^2^Departamento de Psicología, Universidad de Cádiz, Cádiz, Spain

**Keywords:** eating disorders, obesity, emotional processing, food-related images, negative affect

## Abstract

The aim of the present study was to analyze differences in the emotional processing (valence, arousal, and dominance) of food-related information in patients with eating disorders (ED), patients with obesity, and healthy women. Moreover, the mediator role of negative affect and the moderating role of the diagnostic group (ED vs. non-ED) were analyzed. Method: The sample consisted of 94 women (39 with eating disorders, 19 with obesity, and 36 healthy participants). Measures: International Affective Picture System (IAPS) food picture exposure task; Self-Assessment Manikin Analog-Visual Scale (SAM) appraising Arousal, Valence, and Dominance; Eating Attitudes Test (EAT-26); Positive and Negative Affect Schedule (PANAS). Results: Patients with purging symptomatology rated food images as more unpleasant than healthy women. Patients with purging and restrictive eating symptomatology showed higher levels of arousal and less dominance over the emotions experienced, compared to patients with obesity and healthy women. The mediation analysis showed that negative affect mediated the relationship between eating symptomatology (EAT-26) and the Valence of food images, as well as the control over the emotions experienced when viewing food images (Dominance). For the moderation analysis participants were regrouped into two groups (ED patients vs. non-ED patients). The direct relationship between eating symptomatology and food image valence was moderated by the diagnostic group. However, the group did not moderate the direct relationship between the EAT-26 and dominance over experienced emotions, or the indirect effect on eating symptomatology through negative affect. These results show the relevance of negative affect in the emotional processing of food-related information, and they support an eating disorder-disordered eating dimensional perspective.

## Introduction

Eating Disorders (ED) and obesity share multiple biological and environmental risk factors (Haines et al., 2010), and they are associated with maladaptive eating styles, such as restrained eating (i.e., dieting intentions) or emotional eating (Baños et al., 2014), which may be relevant in their development and maintenance (Krug et al., 2013). Moreover, ED and obesity can occur simultaneously or increase in severity over time (da Luz et al., 2018). Individuals with either of these two conditions present altered functioning patterns, including unhealthy behaviors to lose weight or maintain the lost weight (Krug et al., 2013; Segura-Serralta et al., 2020). In this regard, these disorders have increasingly been considered two poles on the same continuum of problems related to eating and weight (Perpiñá and Roncero, 2016; Segura-Serralta et al., 2020). Recent research on the difficulties in their treatment outcomes highlights the role of cognitive, neuropsychological, and emotional factors. Individuals with weight- and eating-related problems show impaired cognitive flexibility and decision-making abilities (Fagundo et al., 2012; Segura-Serralta et al., 2019). The biases in these executive functions are characterized by making decisions based on the short-term consequences (e.g., relief of anxiety), despite long-term negative consequences, and by not learning from previous decisions to modify current behavior (Brogan et al., 2010; Aloi et al., 2015; Perpiñá and Roncero, 2016). In sum, the disorders on the continuum of weight-related problems show a tendency toward decision-making based on immediate rewards (Davis et al., 2010; Aloi et al., 2015; Mallorquí-Bagué et al., 2016).

From an evolutionary standpoint, food is a universally rewarding stimulus that is important for survival (Toepel et al., 2009). Images of food capture the attention (Nummenmaa et al., 2011; Cunningham and Egeth, 2018) and are prioritized during the neural processing (Meule et al., 2013), activating brain areas related to reward, salience, and cognitive control (Dagher, 2012; Tang et al., 2012; Spence et al., 2016). However, food stimuli can be especially rewarding for people with eating symptomatology (Bodell and Keel, 2015; Simon et al., 2016), and they can produce an increase in avoidance behaviors of food-related stimuli (Soussignan et al., 2010; Erdur et al., 2017). On the one hand, patients with binge-eating symptomatology rate food as more enjoyable (Drobes et al., 2001), interesting, exciting (Mauler et al., 2006), and appetizing, favoring food de-inhibition (Carter et al., 2006). However, in turn, these patients rate food stimuli as more aversive and fearful than neutral stimuli (Mauler et al., 2006), due to concerns about the effects of eating on their weight and figure (Giel et al., 2011a), thus favoring dietary restraint. These ambivalent (approach-avoidance) responses to food may indicate that food is processed as a threat to the achievement or maintenance of the ideal of beauty and thinness, apart from being a highly appetitive stimulus (Boutelle et al., 2017). On the other hand, restrictive patients value food more negatively, which increases their anxiety and fear (Giel et al., 2011b), makes them less sensitive to the hedonic and motivational components of food (Racine et al., 2018), and supports their ability to endure long periods of fasting (Friederich et al., 2013). Finally, patients with obesity show increased reactivity to food stimuli (Boswell and Kober, 2016). Taken together, these studies lead to considering negative affect (NA) as an important explanatory factor for biases in the emotional processing of food-related information in patients with ED and weight problems. NA has been postulated as a factor that increases the probability of suffering from eating symptomatology or altered eating (Stice, 2002). The theoretical approach of the affect regulation model (Haedt-Matt and Keel, 2011) indicates that there is a causal relationship between NA and overeating, primarily in restrictive and purgative patients (Tice et al., 2001; Cardi et al., 2015). Similarly, patients with obesity use food as an emotion regulation and stress coping mechanism (Leehr et al., 2015).

**Figure 1:**
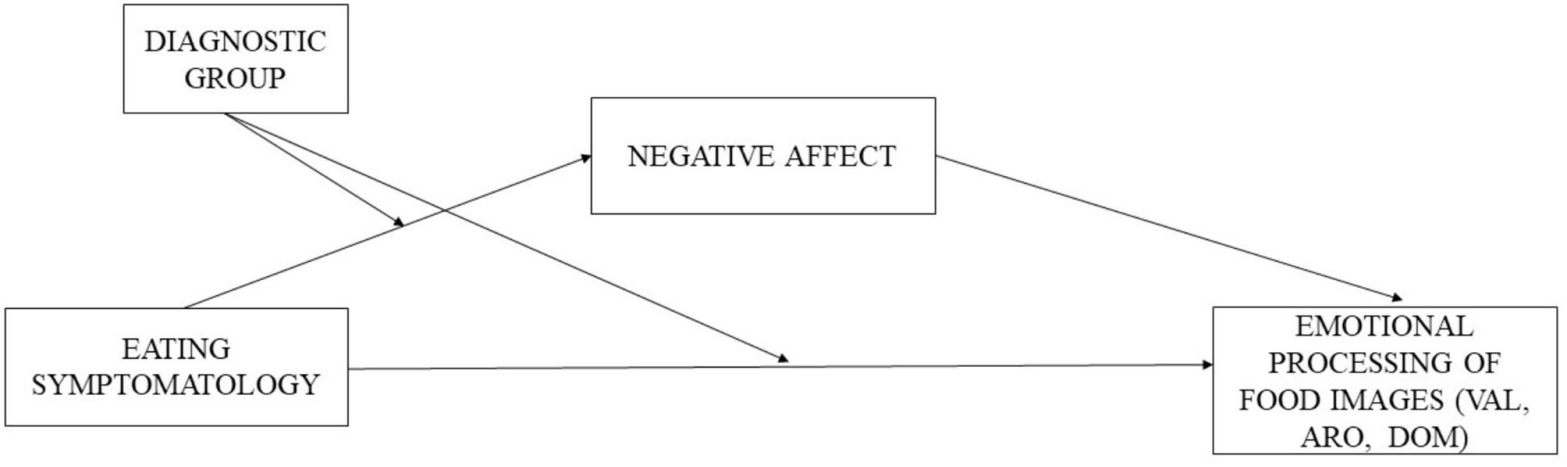
Mediation model with group moderation (ED patients vs. non-ED patients) between the direct effect of eating symptomatology on emotional processing of food images (valence, arousal, and dominance) and between the indirect effect through negative affect. VAL, Valence; ARO, Arousal; DOM, Dominance.

Currently, few studies provide data on the differential emotional processing of food images between patients with ED and patients with obesity, taking into account the role that NA may play in this processing. Knowing the similarities and differences of this emotional processing in ED and obesity may provide significant data that can improve the understanding of the commonalities between them, which in turn will help in their therapeutic approach. Thus, the main aim of the present study was to analyze differences in the emotional processing (valence, arousal, and dominance) of food-related information in patients with ED, patients with obesity, and healthy women. The second aim was to study the relationship between eating symptomatology and emotional processing of food-related information, analyzing the mediator role of NA. Finally, the third aim was to analyze whether the diagnostic group (ED vs. non-ED) moderates the indirect effect between the eating symptomatology through negative affect, and whether the group moderates the direct effect between eating symptomatology and emotional processing (see Figure 1).

## Methods

### Participants and Procedure

The sample consisted of 94 female participants, 39 with a diagnosis of ED, 19 with obesity, and 36 healthy women. In order to simplify the analysis of the composition of the patient groups, and in accordance with the studies carried out so far to find differences between restrictive symptomatology and binge-purge symptomatology, the sample was regrouped according to the symptomatology. Thus, the clinical sample was classified into three groups: restrictive group, formed by patients with restrictive anorexia nervosa or unspecified eating disorder-anorexia nervosa (mean age = 23.48; *SD* = 9.27; *n* = 21); the binge-purge group was formed by patients with purgative anorexia nervosa, purgative bulimia nervosa, or unspecified eating disorder-bulimia nervosa (mean age = 25.50; *SD* = 9.11; *n* = 18); and the obesity group consisted of women with a BMI > 30 (mean age = 46.68; *SD* = 13.44; *n* = 19). The healthy comparison group consisted of women without any mental disorder and a normative weight (BMI = 20–25) (mean age = 30.11; *SD* = 12.34; *n* = 36).

The clinical sample for this study was recruited in three hospitals in the Valencian Community (Spain). The control group was recruited in postgraduate courses at the University of Valencia. To be included in the study, patients with ED could not present psychotic comorbidity or substance abuse, and patients with obesity could not meet the criteria for binge-eating disorder or another mental disorder. The assessment was carried out in two individual sessions. In the first session, the inclusion and exclusion criteria were checked, and the selected International Affective Picture System (IAPS) images were shown to the participants on a laptop computer via the E-PRIME software for stimulus presentation. The study received the approval of the ethics committees of the University of Valencia (H1409824786250) and of each hospital from the National Health System involved in the project. There was no compensation (e.g., economic or course credit) for participating in the study.

### Measures

#### International Affective Picture System

Nine food images (no 2299, no 2702, no 2736, no 6250.2, no 7281, no 7285, no 7410, no 7450 and no 7480) were selected from the International Affective Picture System (IAPS; Lang et al., 2008).

#### Self-Assessment Manikin

We used the Spanish adaptation (Moltó et al., 2013), with a 9-point Likert-type response scale to measure the emotional response in three dimensions rating IAPS images: affective Valence, Dominance, and Arousal. The Spanish adaptation has shown good test-retest reliability: 0.99 for affective valence, 0.97 for dominance, and 0.96 for arousal (*p* ≤ 0.0001) (Lang, 1980; Moltó et al., 2013), and a good level of agreement in the three dimensions (Cohen's Kappas of 0.87, 0.86, and 0.75, respectively; *p* ≤ 0.0001).

#### Eating Attitudes Test

Self-report questionnaire that assesses attitudes toward food and eating symptomatology through 26 items rated on a 6-point Likert-type scale classified as 0 (never, rarely, sometimes), 1 (often), 2 (almost always), or 3 (always). The items are grouped into three factors: dieting or food restriction, bulimic behavior, and preoccupation with food and oral control (Garner and Garfinkel, 1979). The Spanish version adapted by Castro et al. (1991) was used. The internal consistency (Cronbach's α) for the sample in this study was 0.94 for the total scale.

#### Positive and Negative Affect Schedule

Self-report measure that assesses positive and negative affect and is composed of 20 Likert-type items with responses ranging from “not at all” to “extremely” (Watson et al., 1988). The PANAS-PA assesses the ability to engage in pleasant activities, and the PANAS-NA assesses the presence of self-perceived distress. For the present study, the Spanish version by Sandín et al. (1999) was used, employing only the 10 items from the Negative Affect factor (e.g., sad, nervous, or upset). The internal consistency (Cronbach's α) for the sample in this study was 0.93.

### Data Analyses

Spearman correlations were performed between the variables in the proposed model. Then, a Kruskal–Wallis test was conducted between the groups (ED patients with restrictive and purging symptomatology, obesity, and healthy women) on the IAPS scores. Next, using the PROCESS macro for SPSS (Hayes, 2013), three mediation analyses (Model 4, Hayes, 2013) were performed with the whole sample, taking the total score on the EAT-26 as the predictor and the IAPS score (valence, dominance, and arousal) through negative affect (NA) as the mediator. Subsequently, a moderated mediation analysis (Model 8, Hayes, 2013) was performed where the group variable was included as a moderating variable in the relationship between the EAT-26 and the score on the IAPS, and in the relationship between the EAT-26 and negative affect. Indirect effects were calculated using the bootstrapping procedure with 10,000 subsamples. The indirect pathway is significant when the 95% CI does not include the value 0, and so it can be stated that mediation exists.

## Results

### Preliminary Analysis

The associations between the variables studied were analyzed. Positive and statistically significant relationships were found between the total score on the EAT-26 and PANAS-NA (*r* = 0.570), as well as with emotional arousal (IAPS-Arousal) toward food-related images (*r* = 0.210). However, the association was statistically significant and negative between the EAT-26 and the appraisal (IAPS-Valence) of food images (*r* = −0.349), as well as the experienced degree of control over the elicited emotion (IAPS-Dominance) (*r* = −0.483).

### Between-Group Mean Comparisons of the Emotional Response to Food-Related Stimuli

A Kruskal–Wallis test was performed among the four groups of participants (patients with purgative symptomatology, patients with restrictive symptomatology, patients with obesity, and healthy women) on the emotional processing of food-related images. Statistically significant differences were observed for IAPS-Arousal and IAPS-Dominance, and a trend was observed for IAPS-Valence (see Table 1). Specifically, patients with purgative eating symptomatology rated food images more unpleasantly than controls. In addition, patients with purgative and restrictive eating pathology differed from patients with obesity and controls in that they reported a higher level of arousal when visualizing food images. Patients with restrictive eating pathology also differed from the healthy women in IAPS-Dominance. Moreover, patients with purging symptomatology differed from the patients with obesity and healthy women in IAPS-Dominance.

**Table 1:** Kruskal–Wallis test between membership groups on emotional processing score.

**IAPS**	***n***	***M***	**(** ***SD*** **)**	***H***	***df***	***p***	***Post hoc*** **Dunnett (Cohen's** ***d*** **)**
**Valence**							
(1) ED restrictive	21	5.48	1.57	7.66	3	0.05	–
(2) ED purgative	18	5.02	2.27				2 < 4 (0.89)
(3) Obesity	19	5.91	1.00				–
(4) Healthy women	36	6.39	1.02				–
**Arousal**							
(1) ED restrictive	21	5.69	1.36	18.59	3	<0.001	1 > 3 (0.80), 1 > 4 (0.94)
(2) ED purgative	18	5.88	1.23				2 > 3 (0.94), 2 > 4 (1.11)
(3) Obesity	19	4.39	1.87				3 < 1, 3 < 2
(4) Healthy women	36	4.38	1.40				4 < 1, 4 < 2
**Dominance**							
(1) ED restrictive	21	4.78	1.79	29.03	3	<0.001	1 < 4 (0.94)
(2) ED purgative	18	3.74	1.31				2 < 3 (1.51), 2 < 4 (1.78)
(3) Obesity	19	5.90	1.53				3 > 2
(4) Healthy women	36	5.37	1.44				4 > 1, 4 > 2

*IAPS, International Affective Picture System; ED, eating disorders*.

### Mediation Analysis

The results showed that the relationship between the eating psychopathology (EAT-26) and the rating of the food images (IAPS-Valence) was statistically significant across PANAS-NA, resulting in full mediation (completely standardized indirect effect= −0.20; 95% CI [−0.34, −0.08]). In the same way, a full mediation effect was also found between the EAT-26 and the control of emotions experienced when visualizing food images (IAPS-Dominance) through PANAS-NA (completely standardized indirect effect = −0.29; 95% CI [−0.44, −0.17]). Finally, there was no mediation effect in the relationship between the EAT-26 and IAPS-Arousal through PANAS-NA (completely standardized indirect effect = −0.03; 95% CI [−0.09, 0.16]. The results of the three mediation analyses are presented in Table 2.

**Table 2:** Regression coefficients and summary of the three mediation models.

**Variables**	***B*** **^a^**	***SE***	***t***	***CI 95%***	***R*** ^**2**^
Criterion: IAPS-Valence (*Y*)					0.26
PANAS-Negative affect (*M*)	−0.35	0.02	−3.10^*^	[−0.08, −0.02]	
EAT-26 (*X*)	−0.22	0.01	−1.98	[−0.04, 0.01]	
Criterion: IAPS-Dominance (*Y*)					0.37
PANAS-Negative affect (*M*)	−0.50	0.02	−4.83^**^	[−0.12, −0.05]	
EAT-26 (*X*)	−0.16	0.01	−1.54	[−0.04, 0.01]	
Criterion: IAPS-Arousal (*Y*)					0.11
PANAS-Negative affect (*M*)	0.05	0.02	0.40	[−0.03, 0.05]	
EAT-26 (*X*)	0.30	0.01	2.41^*^	[0.01, 0.05]	

*X, Predictor variable; Y, Criterion variable; M, Mediator variable*.

** *p < 0.01*,

* *p < 0.05*.

a *Standardized coefficients*.

### Moderated Mediation Analysis

**Figure 2:**
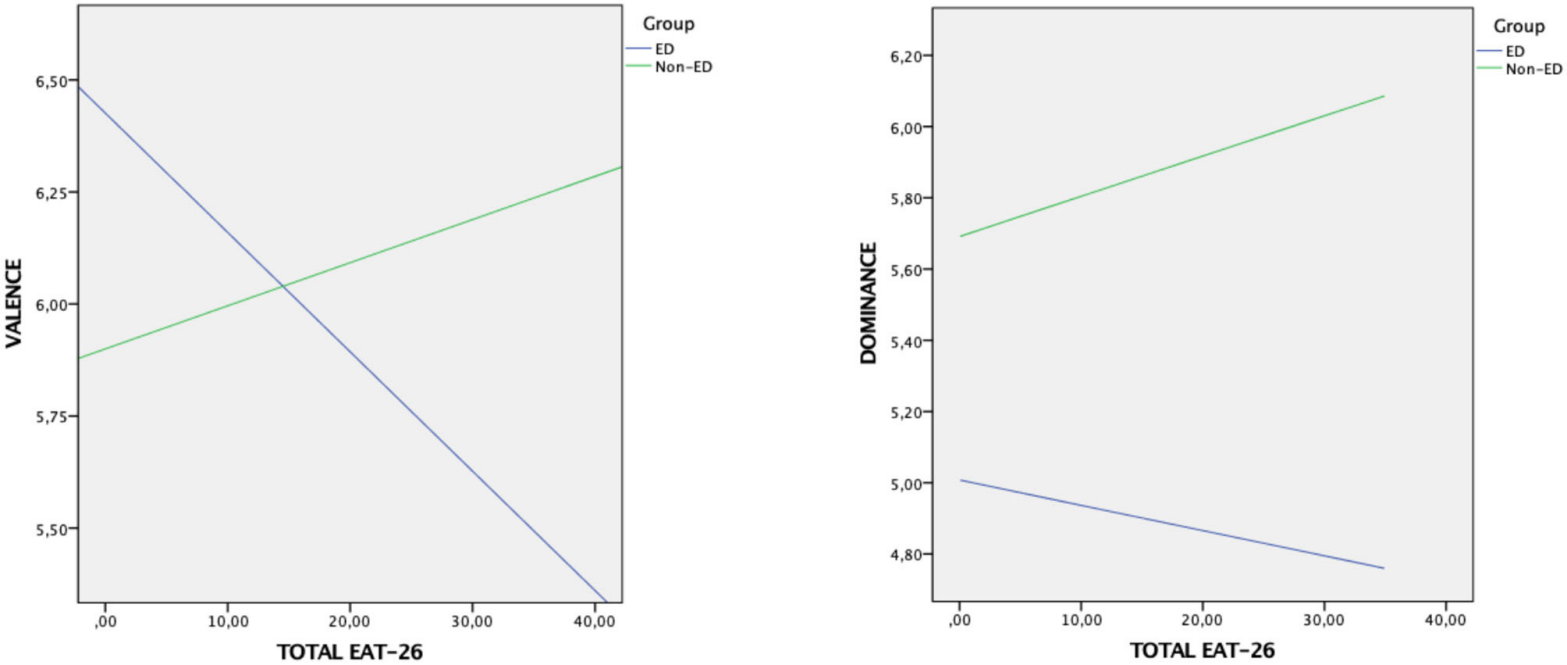
Interaction effect of the moderator variable group on the emotional processing of food images (Valence, Dominance) according to the EAT-26 score.

On the Kruskal–Wallis test, differences were found between ED patients and non-ED patients on the IAPS dimensions. Therefore, the diagnostic group variable (ED patients vs. non-ED patients) was taken as a moderator of the direct effect between the EAT-26 and emotional processing (Valence and Dominance) and as a moderator of the indirect effect between these two variables through PANAS-NA. The group variable moderated the direct relationship between the EAT-26 and IAPS-Valence (conditional direct effect for ED patients vs. non-ED patients: β = −0.03, *SE* = 0.01, *95% CI* [−0.05, −0.01]; non-ED patients: β = 0.01, *SE* = 0.02, *95% CI* [−0.04, 0.06]) (see Figure 2), but it did not moderate the indirect effect (index of Moderated Mediation = −0.01, *SE* = 0.01, *95% CI* [−0.02, 0.01]). With regard to the direct relationship between the EAT-26 and IAPS-Dominance, there was no moderation effect of the group variable (conditional direct effect for ED patients: β = −0.01, *SE* = 0.01, *95% CI* [−0.03, 0.02]; non-ED patients: β = 0.01, *SE* = 0.03, *95% CI* [−0.04, 0.06]) (see Figure 2), and no moderation effect between the EAT-26 and PANAS-NA (moderated mediation index: β = −0.02, *SE* = 0.02, *95% CI* [−0.03, 0.01]).

## Discussion

The main purpose of this study was to analyze the differences in the emotional processing of food-related information in patients with ED, patients with obesity, and healthy women. We found that ED patients presented more dysfunctional emotional processing of food information, experiencing greater arousal (Arousal), less control over their emotions (Dominance), and less attraction to food images (Valence). Overall, these data are consistent with studies showing that ED patients present avoidance reactions to food (Erdur et al., 2017), react with fear and anxiety to these stimuli (Friederich et al., 2006, 2013; Giel et al., 2011b; Steinglass et al., 2012), and rate them as aversive and fearful (Mauler et al., 2006). However, our data differ from other studies reporting that these patients (compared to healthy participants) rate food as more rewarding (Bodell and Keel, 2015; Leehr et al., 2016; Simon et al., 2016), enjoyable (Drobes et al., 2001), and appetizing (Mauler et al., 2006). This ambivalence toward food could be explained by the existence of an approach-avoidance motivational conflict in people with ED (Wilson et al., 2020). Regarding the Valence, our results indicate that patients with binge-purge symptomatology explicitly rate the images as less pleasurable (Racine et al., 2018). These results, in general terms, are in line with those found by previous research indicating that, in these patients, the basic motivational value of food might change (Racine et al., 2018). In addition, patients with binge-purge symptomatology would be more emotionally affected, presenting a high emotional intolerance that leads them to ingest large amounts of food in response to negative emotions (van Strien et al., 2013). Therefore, it is understandable that information related to food, both through images and direct experiences, would produce a loss of dominance, security, or control over the emotions they experience.

Regarding the group of people with obesity, our results indicate that their emotional processing would lie at an intermediate point between patients with ED and healthy women, showing some similarities with both groups. In reality, the presence of particular characteristics in the processing of food-related information has been studied less in obesity than in ED (Castellanos et al., 2009). Nevertheless, studies carried out to date point out that generalized exposure to food stimuli increases physiological reactivity in patients with obesity, influencing their eating behavior and weight gain (Boswell and Kober, 2016). Our results support models based on a transdiagnostic view of disordered eating and ED, validating the entire spectrum of EDs and including obesity (Aloi et al., 2015). Hence, ED and eating problems would be found on the same continuum, with similar difficulties and impairments, making it possible to use similar treatments for both EDs and obesity (Segura-Serralta et al., 2020).

In relation to the second objective, NA mediated the relationship between eating symptomatology (EAT-26) and the emotional processing of food images in terms of Dominance and Valence. However, our results show that, in the relationship between eating symptomatology and Arousal, NA does not seem to play a role. These data are related to a large number of studies indicating that NA predicts the occurrence of ED (Jacobi et al., 2011; Michopoulos et al., 2015; Vannucci et al., 2015), and they support the causal relationship between NA and eating symptomatology, mainly in restrictive and binge-purge patients (Cardi et al., 2015). In a study by Ciscar et al. (2019), the authors observed that all the clinical groups (restrictive patients, binge-purge patients, and patients with obesity) were characterized by high NA and low positive affect. Studies indicate that NA and less cognitive control lead to restrictive or binge eating as a regulatory strategy in the presence of negative emotions (Macht, 2008; Mallorquí-Bagué et al., 2017). In fact, patients with binge-purge symptomatology present higher rates of binge eating and purging on situations characterized by higher NA (Crosby et al., 2009). Something similar occurs in restrictive patients, who seem to show an association between restriction and NA (Engel et al., 2005; Lavender et al., 2016). This makes sense based on the theoretical approaches of the affect regulation model (Haedt-Matt and Keel, 2011), which postulates that NA triggers emotional eating and, at the same time, eating symptomatology is reinforced by the feeling of control over hunger or relief from NA (O'Hara et al., 2015). In patients with obesity, research indicates that obesity is related to the presence of higher NA (Pasco et al., 2013). Loeber et al. (2018) investigated deficits in inhibition of the eating response to food and non-food stimuli in women with obesity. They observed that NA triggered loss of control, concluding that emotional self-control foundered when the women experienced NA (Heatherton and Wagner, 2011; Chester et al., 2016), and that food was used as a strategy to regulate their emotions and cope with stress (Devlin, 2007).

Third, we analyzed whether this relationship between eating symptomatology and NA is moderated by the diagnostic group (ED vs. non-ED). Our results indicate that having a diagnosis of ED influences the assessment of the images, depending on the severity of the eating symptomatology present. That is, patients with greater eating symptomatology gave a worse rating to the food images, but this was not the case in the group of non-patients. Moreover, the relationship between eating symptomatology and NA was not moderated by the diagnostic group, so that this relationship does not depend on the presence of an ED diagnosis. These data seem to point to a dimensional continuum for the relationship between eating symptomatology and negative affect, which is consistent with studies conducted with non-clinical samples showing that participants without ED ate more after experiencing NA (Macht, 2008). Similarly, eating in response to negative emotions is not exclusive to ED and eating problems; populations with depression (Dingemans et al., 2015), anxiety (Dalrymple et al., 2018), and fatigue (Constant et al., 2018) have been observed to use food as an emotional regulation strategy. It appears that people who show a strong sensitivity to food cues along with low emotional control are more likely to overeat (Nederkoorn et al., 2010; Lawrence et al., 2012).

In summary, our results indicate, first, that ED patients have a different emotional processing of food-relevant information compared to healthy women. Their emotional processing is characterized by more negative appraisals, less emotional dominance, and a higher level of arousal, whereas patients with obesity would be in an intermediate position, showing similarities with both ED and healthy women. Second, the results show that NA has a mediator role between eating symptomatology and emotional processing, negatively affecting the appraisal of food stimuli and producing less dominance over the emotions they produce. Third and finally, the ED diagnosis has a decisive influence on the negative appraisal of food images, but the effect of NA on the relationship between eating symptomatology and Valence and Dominance does not depend on the diagnosis.

This study has several limitations. First, it is a cross-sectional study with a proposed tentative model that does not allow us to draw causal inferences. In addition, the groups only contain women because a large percentage of ED patients are women. Moreover, based on our results, it would be interesting to analyze the differences in the emotional processing of food stimuli between patients who are in the recovery phase and those who are not, including some variables that were not controlled, such as the duration of the disease and levels of depression and alexithymia. Future studies should replicate the present analysis with a larger sample of patients with restrictive and purging eating disorders subtypes. Nevertheless, to our knowledge, the present study is the first to compare the emotional processing of food images in a wide variety of ED and disordered eating including obesity, differentiating between restrictive, and binge-purge symptomatology.

## Data Availability Statement

The raw data supporting the conclusions of this article will be made available by the authors, without undue reservation.

## Ethics Statement

The studies involving human participants were reviewed and approved by University of Valencia. The patients/participants provided their written informed consent to participate in this study.

## Author Contributions

CP and MR designed the study. IS prepared the first draft of the manuscript. CS-C performed the statistical analyses. All authors reviewed the manuscript.

## Conflict of Interest

The authors declare that the research was conducted in the absence of any commercial or financial relationships that could be construed as a potential conflict of interest.

## Publisher's Note

All claims expressed in this article are solely those of the authors and do not necessarily represent those of their affiliated organizations, or those of the publisher, the editors and the reviewers. Any product that may be evaluated in this article, or claim that may be made by its manufacturer, is not guaranteed or endorsed by the publisher.
